# Serotonin reuptake inhibitors and its cognitive burden…or relief. A brief review

**DOI:** 10.1192/j.eurpsy.2023.2146

**Published:** 2023-07-19

**Authors:** M. J. Amorim, F. Araújo, P. Passos Perestrelo, M. Maia Marques

**Affiliations:** Departamento de Psiquiatria e Saúde Mental, Unidade Local de Saude do Alto Minho, Viana do Castelo

## Abstract

**Introduction:**

The use of selective serotonin reuptake inhibitors (SSRIs) has increased exponentially and worldwide in the last decade. Taking into account their tolerability and safety profile, they constitute the first line, in all age groups and in polimedicated population, for treatment of depressive, anxious, and phobic disorders, among others. Although safe, especially when compared to first generation antidepressants, they are not totally exempt of adverse effects, and may cause, especially in this context, some degree of cognitive impairment, which may or may not be completely reversible. On the other hand, and despite the controversy related to the subject, some studies suggest a possible protective effect of this pharmacologic class regard the development of cognitive disfunction, which, although not very consistent, should not be neglected.

**Objectives:**

To understand the cognitive impact of short- and long-term use of SSRIs, given the increasing use in an aging, polymedicated population.

**Methods:**

Brief sistematic review of selected articles from Pubmed, Medline and Uptodate databases, with the keywords “*SSRIs*”, “c*ognitive*”, “*impairment*”, “*adverse effects*”, “*dementia*”.

**Results:**

SSRIs are not entirely exempt from cognitive effects, despite their recognized safety profile. Some of the adverse effects typically related to the class, such as hyponatraemia, especially when insidious and silent course, as well as anticholinergic activity (typically associated with first generation antidepressants, but not exclusive) interfere with global functionality and may clinically present as mild cognitive impairment or even major neurocognitive disorders. Furthermore, given their potential to induce or inhibit the cytochrome P450 system, they are significantly implicated in pharmacokinetic drugs interactions that increase cognitive burden associated with other psychotropic drugs.

In the long term, and in certain patient populations, it has been hypothesized that they may exert some protective effect on physiological and pathological cognitive functions decline, by preventing neuronal death, acetylcholine decrease and amoyloidogenesis.

**Conclusions:**

Despite their benign adverse effect profile, when compared with tricyclic antidepressants, SSRIs are not devoid of adverse effects on cognitive domains. However, and despite contradictory and inconsistent results, when well tolerated, SSRIs may confer benefits in terms of preserving global functionality, far beyond the affective symptoms resolution that motivated their introduction in the first place.

**Disclosure of Interest:**

None Declared

